# Effect of angiotensin converting enzyme gene I/D polymorphism in South Indian children with nephrotic syndrome

**DOI:** 10.7555/JBR.32.20150095

**Published:** 2016-03-13

**Authors:** Aravind Selvin Kumar Ramanathan, Balakrishnan Karuppiah, Murali Vijayan, Kamaraj Raju, Dhivakar Mani, Rathika Chinniah, Manikandan Thirunavukkarasu, Padma Malini Ravi, Jeyaram Illiayaraja Krishnan, Prabha Senguttuvan

**Affiliations:** 1. Department of Pediatric Nephrology, Institute of Child Health and Hospital for Children, Chennai, Tamil Nadu 600008, India; 2. Department of Medical Genetics, The Tamil Nadu Dr. M.G.R. Medical University, Chennai, Tamil Nadu 600032, India; 3. Department of Immunology, School of Biological Sciences, Madurai Kamaraj University, Madurai, Tamil Nadu 625021, India; 4. Department of Biotechnology and Genetic Engineering, Bharathidasan University, Tiruchirappalli, Tamil Nadu 620024, India; 5. Department of Clinical Research, Narayana Health City, Bangalore, Karnataka 560099, India; 6. Department of Pediatric Nephrology, Dr. Mehta's Children's Hospital, Chennai, Tamil Nadu 600031, India.

**Keywords:** angiotensin converting enzyme, focal segmental glomerulosclerosis, minimal change disease, nephrotic syndrome

## Abstract

Nephrotic syndrome is one of the most common childhood kidney diseases. It is mostly found in the age group of 2 to 8 years. Around 10%–15% of nephrotic syndrome cases are non-responders of steroid treatment (SRNS). Angiotensin converting enzyme (*ACE*) (I/D) gene association studies are important for detecting kidney disease and herein we assessed the association of *ACE* (I/D) polymorphism with nephrotic syndrome in South Indian children. We recruited 260 nephrotic syndrome (162 boys and 98 girls) and 218 (140 boys and 78 girls) control subjects. *ACE* I/D polymorphism was analyzed by PCR using genotype allele specific primers. In *ACE* (I/D), we did not find significant association for the ungrouped data of nephrotic syndrome children and the control subjects. Kidney biopsies were done in 86 nephrotic syndrome cases (minimal change disease, *n*=51; focal segmental glomerulosclerosis, *n*=27; diffuse mesangial proliferation, *n*=8). We segregated them into the minimal change disease / focal segmental glomerulosclerosis groups and observed that the *ACE* ‘D’ allele was identified with borderline significance in cases of focal segmental glomerulosclerosis and the ‘I’ allele was assessed as having very weak association in cases of minimal change disease. ‘II’ genotype was weakly associated with minimal change disease. Gender specific analysis revealed weak association of ‘ID’ genotype with female nephrotic syndrome in females. Dominant expression of DD genotype was observed in males with nephrotic syndrome. Our finding indicated that *ACE* (I/D) has moderate association with focal segmental glomerulosclerosis. However, due to the limited number of biopsy proven focal segmental glomerulosclerosis subjects enrolled, further studies are required to confirm these results.

## Introduction

Nephrotic syndrome (NS) is a condition where damages are prominent in filtering units of kidney. NS is more predominant in children and commonly exists in the ages of 2 and 8 years. It seems to affect boys more often than girls^[1^–^2]^. The annual incidence of NS in most countries is estimated to range from 2 to 7 new cases per 100,000 children. The annual incidence of NS in US, European, Africo-Caribbean and Asian children were 2, 2.6, 3.4 and 16.9 per 100,000 children respectively and the cumulative prevalence was about 16 per 100,000^[1^–^4]^. A geographically based epidemiological study of NS suggested that Asian children have higher incidence; mainly, it was higher in lower socio-economic groups^[5^–^6]^. Ethnic origin may play a major role of the histological modification and response to immunosuppressive treatment. Based on immunosuppressive treatment, NS is mainly divided into steroid sensitive, steroid dependent and steroid resistant. The histological evaluation of renal tissues in nephrotic cases has three main categories: minimal change disease (MCD), focal segmental glomerulosclerosis (FSGS) and diffuse mesangial proliferation (DMP). MCD is idiopathic, with no change in the number of podocytes and it mostly responds to steroid treatment (remission). FSGS is a severe form which decreases the number of podocytes and does not respond to steroid treatment. Finally, DMP is also a severe form of nephrotic which is rarely seen.

Genetic factors have been suggested to be important in determining the progression of NS. Recently, well documented evidences indicate that the renin-angiotensin system (RAS) is involved/implicated in pathogenesis of kidney disease^[7^–^8]^. Angiotensin converting enzyme (ACE) is an important enzyme of RAS. The stability of plasma ACE levels, combined with marked inter individual differences and familial clustering of plasma ACE levels suggested its regulation to be under major gene control. Genes that are involved in the RAS system are functional candidates/close link with these phenotypes, it would be the prognostic factors of renal damage such as diabetic nephropathy, Immunoglobulin A (IgA) nephropathy^[7]^, systemic lupus erythematosus(SLE)^[9]^ and/or lupus nephritis and finally lead to development of end stage renal disease. Some of the existing reports from *ACE* polymorphism identified the presence of ‘D’ allele is indicated for the risk of cardiovascular^[10]^ and kidney damage^[11]^. *ACE* DD genotype is responsible for hypertension^[8]^, diabetes mellitus^[12]^, IgA nephropathy^[7]^, renal artery stenosis^[7]^, cardiomyopathies^[13]^and carotid atherosclerosis^[14]^. However, some previous studies evaluating/identify and proven relationship between steroid sensitive/FSGS and *ACE* gene polymorphism of NS in different ethnic populations^[15^–^16]^. *ACE*-II genotype was more frequent in steroid sensitive nephrotic syndrome (SSNS) patients as compared to controls in North India^[17]^, Malaysia^[18]^. DD genotype with NS has been reported from Taiwan Province (China)^[19]^, Egypt^[20]^. However, the DD genotype was associated only with FSGS-SRNS in the Kuwaiti Arab children^[21]^. In contrast to the previous studies, no such association of *ACE* gene polymorphism was found in Swiss children^[22]^. Hence, we analysed the distribution of *ACE* (I/D) gene polymorphism of NS [SSNS/steroid resistant nephrotic syndrome (SRNS)] in South Indian children between the age of 2 and 12 years old.

## Subjects and methods

### Study subjects

In the present study, we recruited 260 (mean age of 7.17±3.58) NS cases from a single center; 218 control subjects (mean age of 10.5±1.07) were recruited. Our institutional ethical committee approved the study and written informed consent was obtained from subject’s parents. All enrolled children were born in South India, were of ethnically South Indian ancestry and belonged to the lower and middle classes. Nephrotic cases were sporadic and it was noted that 85 enrolled (nephrotic 58; control 27) subjects were born from second degree consanguineous parents. The enrolled subjects were between the ages of 2 and 12 years old. The inclusion criterion was the clinical presentation of NS. The diagnosis of NS was based on the presence of edema, urinary protein excretion ≥ 40 mg/(m^2^·hour), spot albumin to creatinine ratio>2 mg, and hypoalbuminemia<2.5 g/dL. All the nephrotic cases received the standard steroid therapy and were classified into two categories on the basis of their clinical responses towards steroids: SSNS group and SRNS group. SSNS is defined as (remission) stratified into proteinuria negative to trace for three consecutive days or urine protein excretion<4 mg/(m^2^·hour). SRNS is defined as failure to achieve remission after 4 week of daily oral prednisolone at a dose of 2 mg/(kg·day). Children with NS and a history of positive HIV, HbsAg were excluded from the study. The control group consists of unrelated healthy individuals with no history of kidney disease/hypertension.

### DNA isolation and *ACE* (I/D) genotyping

Genomic DNA was isolated from 2 mL of blood using the standard salting-out method. The 16^th^ intron of the polymorphic *ACE* (I/D) gene was amplified by PCR. The primers (forward/reverse) 5’- TGGAGACCACTCCCATCCTTTCT-3’ and 5’- GATGTGGCCATCACATTCGTCACGAT-3’ were used to amplify the region of intron 16 which produced a 287-bp insertion/deletion polymorphism. PCR reaction was performed in a final volume of 12 μL containing 7.64 μL of milli Q water, 3 μL of genomic DNA (200 ng/mL), 0.2 μL of 5U *Taq* polymerase (Genet Bio, Korea), 1.2 μL of 10 × PCR buffer, 0.24 μL of 10 mmol/L dNTP (Cinna Gen, Iran), 0.36 μL each of forward and reverse primer (10 mmol/L). PCR amplification was carried with an initial denaturation at 94 °C for 5 minutes, followed by 30 cycles of denaturation at 94 °C for 2 minutes, annealing at 58 °C for 1 minute and extension at 72 °C for 1 minute and a final extension at 72 °C for 5 (Agilent, USA; model no: G8800A). Amplified products were detected on a 2% agarose gel containing 0.5 μg/mL of Ethidium Bromide. The amplicon containing the insertion allele (I) was visible as a band at approximately 490 bp; while deletion (D) at 190 bp; and 190-bp and 490-bp PCR products for ID heterozygotes. Due to preferential amplification of the D allele, it was possible that ID heterozygotes might be mistyped as DD homozygotes. Therefore, in order to increase the specificity of DD genotyping, all samples identified as DD after initial amplification were reconfirmed with the second PCR containing an insertion specific primers.

### Statistical analysis

The statistical analysis was performed by STATA 11.1 (College Station, TX, USA). The continuous variables of age, serum creatinine, protein and albumin and cholesterol level were expressed as mean and standard deviation. *ACE* (I/D) genotype and allele frequencies were compared between the cases and controls. Odds ratio with 95% confidence interval expressed as genotype and allele frequencies. Genotype and allele frequencies distribution were expressed as frequency and percentage. Hardy-Weinberg equilibrium (HWE) for *ACE* genotype was tested by Chi square test in each group. Distribution of *ACE* (I/D) polymorphism dominance and recessive model were expressed. Statistically significant accepted value was *P*<0.05.

## Results

The clinical and demographic data of the nephrotic cases are presented in *Table 1*. There were no statistically significant differences in the distribution of *ACE* (I/D) gene polymorphism between the NS cases and controls (*Table 2*). The data were stratified based on steroid treatment response in NS cases. The differences in the frequencies for genotypes or alleles for SSNS and SRNS were found to be statistically insignificant. We segregated NS biopsy cases into the MCD and FSGS groups. We found II genotype frequency was observed in 29.41% (15/51) of the MCD cases and in 11.11% (3/27) FSGS cases. This association was estimated as [OR(95%CI)=3.33(0.80–19.61); *P*=0.06] (*Table 3*). The frequency of DD genotype was 25.9% (7/27) in FSGS, but 11.7% (6/51) in MCD. The ‘I’ allele and ‘D’ allele were found to be weakly associated (both had borderline significance) with MCD and FSGS, respectively. Additionally, in 8 DMP cases, II and ID genotype frequencies were 50% (4/8) and 50% (4/8), respectively, no cases had DD genotype. However, when we segregated the groups into male and female populations of nephrotic cases and controls, we could not found a significant association for II, ID and DD genotype in males. In the female groups, ID genotype was slightly more prevalent (59.1%) in the nephrotic group than the control (51.2%) which showed a weak association [OR (95%CI) =1.74 (0.93–3.24); *P*=0.06] (*Table 4*). Logistic regression analysis of the genetic model was carried out in dominant [(II+ID) *vs*. DD], recessive [II *vs*. (DD+ID)], additive (II *vs.*DD) and co-dominant [ID *vs*. (DD+II)] for male, female and pooled NS and control subjects. The result of the dominant model, which revealed a significant association, was estimated [OR(95%CI)=0.14 (0.07–0.25); *P*=0.001] in male nephrotic cases (*Table 5*). Further, we calculated the degree of dominance (h) test to find out the deviation of heterozygous state from the risk of disease. The analysis revealed that the degree of dominance was<1 from the *ACE* (I/D) variant and NS in South Indian children.

**Tab.1:** Clinical and demographic details of patients

Variables	Value
NS (M,F)	260 (162,98)
SSNS (M,F)	107 (68,39)
SRNS (M,F)	153 (94,59)
Age (years)^ψ^	7.17±3.58
Serum creatinine (mg/dL)^ψ^	0.78±0.43
Serum protein (mg/dL)^ψ^	4.73±1.04
Serum albumin (mg/dL)^ψ^	2.27±0.72
Serum cholesterol (mg/dL)^ψ^	338±125
Biopsy	
MCD/FSGS/DMP	51/27/8

^ψ^ (mean±SD); SSNS: steroid sensitive nephrotic syndrome; SRNS: steroid resistant nephrotic syndrome; MCD: minimal change nephrotic; FSGS: focal segmental glomerulosclerosis; DMP: diffuse mesangial proliferation

**Tab.2:** Distribution of *ACE* (I/D) genotype and allele frequencies in SSNS/SRNS patients and control subjects

Genotype/Allele	Control [*n*(%)] (*n*=219)	NS [*n*(%)] (*n*=260)	OR (95% CI)	χ^*2*^	*P*-value
II	63 (28.76)	76 (29.23)	1.02 (0.67–1.55)	0.01	0.91
ID	130 (59.36)	151 (58.07)	0.94 (0.64–1.39)	0.08	0.77
DD	26 (11.87)	33 (12.69)	1.07 (0.60–1.93)	0.07	0.78
I	256 (58.44)	303 (58.26)	0.99 (0.76–1.29)	0	0.95
D	182 (41.55)	217 (41.73)	1.00 (0.77–1.31)	0	0.95

SSNS: steroid sensitive nephrotic syndrome; SRNS: steroid resistant nephrotic syndrome

**Tab.3:** Distribution of *ACE* (I/D) genotype frequencies in MCD and FSGS subjects

Genotype	MCD [*n*(%)] (*n*=51)	FSGS [*n*(%)] (*n*=27)	OR (95% CI)	χ^*2*^	*P*-value
II	15 (29.5)	3 (11.11)	3.33 (0.80–19.61)	3.33	0.06
ID	30 (58.8)	17 (63.0)	0.84 (0.28–2.42)	0.13	0.72
DD	6 (11.7)	7 (26.0)	0.38 (0.09–1.53)	2.55	0.11
I	60 (58.8)	23 (42.6)	1.92 (0.94–3.97)	3.74	0.05
D	42 (41.2)	31 (57.4)	0.52 (0.25–1.07)	3.74	0.05

MCD: minimal change nephrotic; FSGS: focal segmental glomerulosclerosis

**Tab.4:** Distribution of *ACE* (I/D) genotype and allele frequencies in nephrotic syndrome patients and control stratified by gender wise

Gender	Genotype/ Allele	NS [*n*(%)] (*n*=260)	Control [*n*(%)] (*n*=219)	OR (95% CI)	χ^*2*^	*P*-value
Male	II	48 (29.6)	36 (25.6)	1.34 (0.79–2.30)	1.33	0.24
	ID	93 (57.4)	90 (63.8)	0.76 (0.47–1.25)	1.30	0.25
	DD	21 (12.9)	15 (10.6)	1.25 (0.59–2.73)	0.39	0.53
	I	189 (58.3)	162 (57.4)	1.04 (0.74–1.45)	0.05	0.82
	D	135 (41.7)	120 (42.5)	0.96 (0.69–1.35)	0.05	0.82
Female	II	28 (28.6)	27 (34.6)	0.76 (0.38–1.51)	0.74	0.39
	ID	58 (59.2)	40 (51.2)	1.74 (0.93–3.24)	3.51	0.06
	DD	12 (12.2)	11 (14.1)	0.85 (0.32–2.27)	0.13	0.71
	I	114 (58.2)	94 (60.2)	0.92 (0.48–1.44)	0.16	0.69
	D	82 (41.8)	62 (39.7)	1.07 (0.68–1.68)	0.16	0.69

**Tab.5:** Risk factor analysis of *ACE* genotypic model

Genotypic model	NS Pooled (*n*=260)^a^ Male (*n*=162)^b^ Female (*n*=98)^c^	Control Pooled (*n*=218)^a^ Male (*n*=140)^b^ Female (*n*=78)^c^	OR (95% CI)	χ^*2*^	*P*-value
II+ID *vs*. DD^╪^	227/33	193/26	0.93 (0.51–1.66)	0.07	0.785
	141/21	126/15	0.14 (0.07–0.25)	51.8	0.001
	86/12	67/11	1.18 (0.44–3.11)	0.13	0.716
II *vs*. ID+DD^ψ^	76/184	63/156	1.02 (0.68–1.55)	0.01	0.911
	48/114	36/105	1.23 (0.72–2.11)	0.63	0.427
	28/70	27/51	0.76 (0.38–1.51)	0.74	0.390
ID *vs*. DD+II^ρ^	151/109	130/89	0.95 (0.65–1.39)	0.08	0.776
	93/69	90/51	0.76 (0.47–1.25)	1.30	0.254
	58/40	40/38	1.38 (0.72–2.62)	1.10	0.294
II *vs*. DD^€^	76/33	63/26	0.95 (0.49–1.83)	0.03	0.871
	48/21	36/15	0.95 (0.40–2.25)	0.01	0.904
	28/12	27/11	0.95 (0.32–2.81)	0.10	0.919

^╪^Dominant ; ^ψ^Recessive; ^ρ^Co-dominant ; ^€^Additive; ^a^Pooled; ^b^Males; ^c^Females

## Discussion

ACE is a glycoprotein and its stability in plasma, combined with marked inter-individual differences and the familial clustering of plasma ACE levels, suggests its regulation is under the control of a major gene. *ACE* I/D polymorphism was originally thought to account for varying degrees of phenotypic expression in circulation. The generation of Ang II depends on *ACE*. All nephrotic patients ultimately received the suggested treatment, with the synergic combination of *ACE* inhibitor and Ang II receptor blockers, as they decrease the proteinuria. Thus, it reduces the glomerular capillary pressure in addition to altering the glomerular permeability. Recently, Type 2 diabetes patients with nephropathy (proteinuria>150 mg/24 hours) treated with standard *ACE*I therapy found progressive reduction of the proteinuria during 3–6 months study period^[21]^. Effects of Ang II increased systemic and glomerular blood pressure, leading to tubule-interstitial fibrosis and glomerulosclerosis; finally progressed to loss of kidney function. DD homozygous or D allele is associated with elevated circulating and tissue *ACE* activity compared to I allele. Several studies have found that D allele is an independent risk factor for hypertension, diabetic, IgA nephropathy, congenital renal malformations and NS^[16^,^20^,^24]^.

In the present study, we observed that *ACE* (I/D) allelic distribution was not elevated in the control group when compared to the different subsets of nephrotic (SSNS/SRNS) cases, which is in agreement with previous studies from different ethnic populations such as Swedish and Egyptian^[22^,^25^–^27]^. On the contrary, the *ACE*-II genotype was found more frequent in SSNS in North Indian and Pakistani children^[17^,^28]^. However, DD genotype was higher in north Indian population^[15]^ which also coincided with the Egyptein^[25]^, Turkish^[29]^ and Taiwanese^[19]^ nephrotic children. Therefore, the *ACE* (I/D) polymorphism and its linkage with NS in different ethnic populations are contentious.

Podocyte loss is the main reason behind the development of FSGS. According to the histopathological condition, NS is distinguishable notably as MCD and FSGS. The pathogenesis of MCD has not been clear so far; but there are evidences of immune dysfunction^[30]^. Most MCD cases were in remission, but a very few of them was advanced to CKD. In our center, some of the SRNS cases had a slow progression, while most have fast progressions to end-stage renal disease. It seems that genetic markers/polymorphism plays a susceptible/protective role in the disease mechanism^[31]^. Moreover, existing studies have identified the genetic markers and their linkage to the progression of kidney disease. Firstly, a study on PcKO mice, the *Atg*5 gene (functional block of autophagy) was associated with slow progression of podocyte degeneration and then to glomerulosclerosis^[32]^. Secondly, the *ACE* II-A9570G, *ACE*-ID/DD and/or AGT-M235T polymorphism were associated with an increased risk factor for arterial hypertension in FSGS with fast progression to chronic kidney disease (CKD)^[16^,^31^–^34]^. In the present study, FSGS cases have ‘D’ allele association, which showed substantial persistence of the ‘I’ allele. However, the incidence of II genotype cases (3/27) was less dominant in FSGS. All three ‘II’ genotype FSGS cases had late onset of nephrotic below 10 years of age had slow progression to end-stage renal disease. In MCD cases, the ‘I’ allele showed strong association and the II genotype a weak association with very few of them having DD genotype (11.7%). In this manner MCD-DD genotype may play a role in the slow progression of MCD to FSGS. However, a second biopsy is required to confirm the conversion of MCD to FSGS. In one study from Kuwaiti, in Arab-Jewish nephrotic children the DD genotype was associated with FSGS cases^[21]^. In contrast, one meta-analysis study showed DD genotype/ ‘D’ allele is associated with MCD and II genotype played a protective role^[35]^. In our study, the frequency of ID genotype was slightly increased in SSNS 32.71% (35/107) compared to SRNS 26.79% (41/153) but it was not statistically significant. Moreover, a large number of biopsy proven MCD/FSGS cases may be recommended along with routine follow-up for better interpretation of the role of the *ACE* ID/DD genotype.

In childhood, a preponderance of nephrotic in males is well-established. In an experimental rat model, an inactivating mutation in *ACE* resulted in lower ACE protein, compared with wild type rats. In female rats, a low level of ACE protein did not affect the blood pressure, but male rats were protected from hypertension^[36]^. In gender based study, *ACE* DD genotype was associated with hypertension in males^[37]^. Further, a large study (the Suita Study, Japan) which enrolled 14,200 individuals suggested a unique sex-specific effect of *ACE* on hypertension. In addition, a meta-analysis study involving Asian males with hypertension revealed significant association between *ACE* (I/D) polymorphism and CKD risk^[37]^. On the other hand SLE in females (analysis of 644 families) was proven with *ACE* association^[9]^. In various studies, established *ACE* (I/D) polymorphism and gender based disease association have been documented in different ethnic populations. Lin *et al*. stated that *ACE* (I/D) polymorphism and gender dependent effect have been observed very commonly among different populations. So, *ACE* locus is a sex-specific candidate gene/association for different diseases. In the present study, the subjects were segregated, gender-wise. The gender-wise comparison results revealed that female nephrotic children had weak association of ‘ID’ [OR (95%CI) =1.74 (0.93–3.24); *P*=0.06]. *ACE* ID genotype present in females with NS was 59.1% (58/98) and the control was 51.2% (40/78). In females, we observed ID genotype (subset of nephrotic) MCD, FSGS, DMP and pooled were 66.6% (12/18), 63.6% (7/11), 60% (3/5) and 64.7% (22/34) respectively. We conclude the ID genotype frequency was significantly increased in the NS and subset, compared to control females.

Finally, we found the moderate significant association between the *ACE* ‘D’ allele and FSGS cases. The study results are positive and suggest that future studies should recruit a larger number of biopsy proven FSGS cases from different geographical regions for a better understanding of NS.
